# Listening Effort Measured With Pupillometry in Cochlear Implant Users Depends on Sound Level, But Not on the Signal to Noise Ratio When Using the Matrix Test

**DOI:** 10.1097/AUD.0000000000001529

**Published:** 2024-06-18

**Authors:** Hendrik Christiaan Stronks, Annemijn Laura Tops, Kwong Wing Quach, Jeroen Johannes Briaire, Johan Hubertus Maria Frijns

**Affiliations:** 1 Department of Otorhinolaryngology and Head & Neck surgery, Leiden University Medical Center, Leiden, the Netherlands; 2 Leiden Institute for Brain and Cognition, Leiden, the Netherlands; 3 Department of Bioelectronics, Delft University of Technology, Delft, the Netherlands.

**Keywords:** Cochlear implants, Listening effort, Pupillometry, Sensorineural hearing loss, Speech intelligibility

## Abstract

**Objectives:**

We investigated whether listening effort is dependent on task difficulty for cochlear implant (CI) users when using the Matrix speech-in-noise test. To this end, we measured peak pupil dilation (PPD) at a wide range of signal to noise ratios (SNR) by systematically changing the noise level at a constant speech level, and vice versa.

**Design:**

A group of mostly elderly CI users performed the Dutch/Flemish Matrix test in quiet and in multitalker babble at different SNRs. SNRs were set relative to the speech-recognition threshold (SRT), namely at SRT, and 5 and 10 dB above SRT (0 dB, +5 dB, and +10 dB re SRT). The latter 2 conditions were obtained by either varying speech level (at a fixed noise level of 60 dBA) or by varying noise level (with a fixed speech level). We compared these PPDs with those of a group of typical hearing (TH) listeners. In addition, listening effort was assessed with subjective ratings on a Likert scale.

**Results:**

PPD for the CI group did not significantly depend on SNR, whereas SNR significantly affected PPDs for TH listeners. Subjective effort ratings depended significantly on SNR for both groups. For CI users, PPDs were significantly larger, and effort was rated higher when speech was varied, and noise was fixed for CI users. By contrast, for TH listeners effort ratings were significantly higher and performance scores lower when noise was varied, and speech was fixed.

**Conclusions:**

The lack of a significant effect of varying SNR on PPD suggests that the Matrix test may not be a feasible speech test for measuring listening effort with pupillometric measures for CI users. A rating test appeared more promising in this population, corroborating earlier reports that subjective measures may reflect different dimensions of listening effort than pupil dilation. Establishing the SNR by varying speech or noise level can have subtle, but significant effects on measures of listening effort, and these effects can differ between TH listeners and CI users.

## INTRODUCTION

Cochlear implants (CIs) are neuroprosthetic devices surgically implanted in the inner ear and the treatment of choice for severe to profound sensorineural hearing loss. A CI electrically stimulates the auditory nerve, bypassing the degenerate hair cells in the cochlea (Naples & Ruckenstein 2020). These devices allow for good speech recognition in quiet listening conditions for many individuals. However, background noise reduces speech recognition so that hearing in noisy environments is substantially worse for CI users than for typical hearing (TH) listeners (Cullington & Zeng 2008). The act of listening thus is cognitively demanding for CI users, particularly in adverse listening conditions (Perreau et al. 2017), so that listening to speech can result in higher levels of listening effort and fatigue (Hicks & Tharpe 2002; McGarrigle et al. 2014). In clinical practice, hearing abilities of CI users are most often evaluated in terms of hearing function, but including measures of listening effort and fatigue in these assessments could offer a more comprehensive evaluation of a hearing disability (Humes 1999). For this reason, it is important to measure listening effort using subjective or physiological means (Winn et al. 2018b).

Listening effort is the mental exertion required to understand auditory messages (McGarrigle et al. 2014) and reflects the amount of cognitive resources, such as memory, executive function, and attention, needed for a listening task (Zekveld et al. 2018). Pupillometry is commonly used as an objective measure of listening effort (Winn et al. 2018b), and is based on the observation that pupil size correlates with the magnitude of listening effort (Piquado et al. 2010). Zekveld et al. (2010) showed that pupil dilation increases with decreasing speech intelligibility for TH listeners, concluding that pupillometry can objectively measure listening effort during speech recognition in difficult listening conditions. Pupillometry also has been extensively applied for hearing-impaired people and hearing aid users to characterize listening effort (for a review, see Ohlenforst et al. 2017). Investigations of listening effort for CI users are gaining increased attention, leading to recent studies using pupillometry for this purpose (Winn 2016; Russo et al. 2020; Stronks et al. 2021a; Winn & Teece 2021, 2022).

Listening effort can also be measured subjectively through Likert scale ratings of the effort expended in recognizing speech material (Zekveld et al. 2010; Stronks et al. 2021a). Subjective ratings and objective measures are thought to assess different aspects of listening effort and mental fatigue (Hornsby 2013). It has been shown that subjective ratings are biased and tend to reflect performance more than effort (Moore & Picou 2018). Performing objective measurements (here: pupillometry) may help to disentangle these aspects and give a more reliable estimate of listening effort than subjective measures (here: ratings on a Likert scale).

At present, assessment of the effectiveness of speech enhancement algorithms for CIs is focused on speech recognition performance. However, hearing outcome is a multifaceted measure that also includes listening effort, perceived sound quality, and satisfaction rates (Humes 1999). Most published studies of listening effort as a function of speech intelligibility have been performed for populations other than CI users, such as hearing aid wearers (Ohlenforst et al. 2017). The relatively few investigations with CI users often compared them with other populations, usually TH participants (Steel et al. 2015; Winn 2016; Winn & Moore 2018a; Wagner et al. 2019), and did not directly assess the within-subject effect of signal to noise ratio (SNR) on listening effort. In two studies that investigated the relation between task difficulty and listening effort for CI users, one showed a significant effect of varying speech level on subjective ratings, but not on pupil dilation (Stronks et al. 2021a). The other reported an effect of SNR on pupil responses, but did not provide statistical analyses to support this finding (Kan 2017). We know of only one study (Russo et al. 2020) that reported a significant effect of listening condition on pupillometric measures within a group of CI users. The authors reported larger overall pupil diameters when listening in noise than in quiet; however, data presented in their Figure 3 indicate that increased pupil response in noise resulted mainly from a large dilation at noise onset and less so from subsequently presented speech. The authors did not quantitatively compare dilations with noise-only as baseline. If our interpretation is correct, the findings might show a dependence of the pupillary response on noise level and not on SNR.

To investigate whether listening effort depends on speech intelligibility for CI users, we assessed the effects of SNR on objective and subjective measures of listening effort in a speech-in-noise listening task. To this end, we performed pupillometry and measured subjectively perceived listening effort on a Likert scale at a broad range of SNRs. The findings were compared with a group of predominantly young, TH listeners serving as controls.

We systematically varied the noise level while keeping the speech level constant or varied the speech at a constant noise level to obtain the different SNRs. This allowed us to investigate whether noise-varied or speech-varied SNRs have different effects on listening effort. This research goal was formulated based on the finding that hearing-impaired listeners show a higher sensitivity to noise than TH people do (Aniansson et al. 1983). In effect, noise causes more stress for hearing-impaired listeners, higher annoyance ratings, and increased fatigue (Jahncke & Halin 2012).

## MATERIALS AND METHODS

### Participants

This prospective crossover study included 23 CI users (22 unilateral and 1 bilateral) recruited at the Leiden University Medical Center, and 18 TH control participants recruited mainly from within our department. CI users were implanted with Advanced Bionics devices (Valencia, CA, USA). Seven CI users reported eye disorders, namely Usher syndrome (n = 1), vision loss after meningitis (n = 1), exudative diabetic retinopathy (n = 1), and cataract extraction surgery (n = 4). Their pupil responses appeared normal; eyes with disorders had an average pupil dilation of 12.4 ± 5.6% compared with 12.1 ± 8.2% for the entire CI group, and none of them represented the upper or lower extreme of the response magnitude range. Eyes with morbidities did not result in increased loss of data; each eye with a disorder had 18 trials of 20 included on average, whereas this was 15 of 20 in the CI group as a whole. Any contralateral hearing devices were taken off. Contralateral ears were not plugged. The 18 TH participants had average audiometric pure-tone thresholds across 500 to 4000 Hz (PT_A500-4000_) of 20 dB HL or better in both ears. The CI recipients in the study were older overall (mean 64.3 years, SD 15.6) than the TH controls (mean 33.1, SD 14.3). The demographic data for the CI group are shown in Table 1. Informed consent was obtained from all participants before testing was commenced. This study was approved by the medical ethical committee (IRB) of Leiden, Den Haag, Delft (METC LDD, application numbers P02.106 and P18.177) and adhered to the tenets of Helsinki (World Medical Association 2013).

**TABLE 1.** Demographics of CI users (N = 23)

ID	Age (y)	HL (y)	CI (y)	CVC (%)	PTA (dB HL)	Implant	Etiology	Eye comorbidity
CS09	66	17	4.3	89	107	MS	Progressive, otosclerosis	
CS10	82	5	3.8	82	63	MS	Sudden deafness	
CS11/SR05	49 50	0	3.1 3.8	82	18	1j	Meningitis	
CS12/ SR06	64 65	15	1.1 2.2	95	68	MS	Progressive, unknown	
CS13/ SR07	21 21	20	0.9 1.5	96	87	MS	Progressive, congenital, unknown	
CS14	69	15	1.3	88	82	MS	Progressive, familial	
CS15	67	24	1.5	91	75	MS	Meniere, progressive	
CS16	70	5	1.1	88	100	MS	Familial, progressive	Cataract surgery, scleritis (Cogan syndrome)
CS17	83	12	1.4	84	83	MS	Progressive, unknown	Cataract surgery
CS18	55	12	1.5	90	63	MS	Progressive, congenital?	
SR03	68	14	4.1	86	77	MS	DFNA22 progressive	
SR04	71	34	6.3	92	93	MS	Otosclerosis	
SR08	75	5	3.2	92	68	MS	DFNA9	
SR09	68	2	5.5	90	80	MS	DFNA9	Exudative diabetic retinopathy
SR10	74	15	4.9	86	63	MS	Progressive familial?	Cataract
SR11	52	?	16.8	98	-	CII/1j	Meningitis	Vision loss
SR12	62	?	7.7	93	112	1j	Usher syndrome type 2	Usher syndrome
SR13	82	6	5.0	91	78	MS	Progressive, unknown	
SR14	64	3	5.0	89	75	MS	DFNA9	Cataract surgery, dry eye syndrome
SR15	78	12	7.6	86	98	1j	Progressive, unknown	
SR16	63	7	4.3	96	58	1j	DFNA9	
SR17	61	1	4.8	95	127	MS	Otosclerosis	
SR18	79	26	3.4	83	87	MS	Progressive, unknown	
Min	21	0	0.9	82	18			
Max	83	34	16.8	98	127			
Mean Total	64	12	4	90	82		N = 7	

Three CI users were tested with both 4 sec (CS) and 8 sec baseline and (SR) and received two codes. N = 23 participants with 24 CIs (SR11 was a bilateral user with a CII and 1j implant).

1j: HiFocus 1j Electrode; CI: experience with cochlear implant (y); CII: Clarion CII Bionic Ear system; CVC: consonant-verb-consonant score (% phonemes correct); DFNA: autosomal dominant non-syndromic sensorineural hearing loss (“deafness”); DFNA9: DFNA due to mutation in COCH gene; DFNA22: DFNA mutation in the MYO6 gene; HL: duration of hearing loss (y); MS: HiFocus Mid-Scala electrode; PTA: average pure-tone audiometric threshold at 500, 1000, and 2000 Hz (dB hearing level).

published online ahead of print June 18, 2024

### Test Environment

**Fig. 1.**
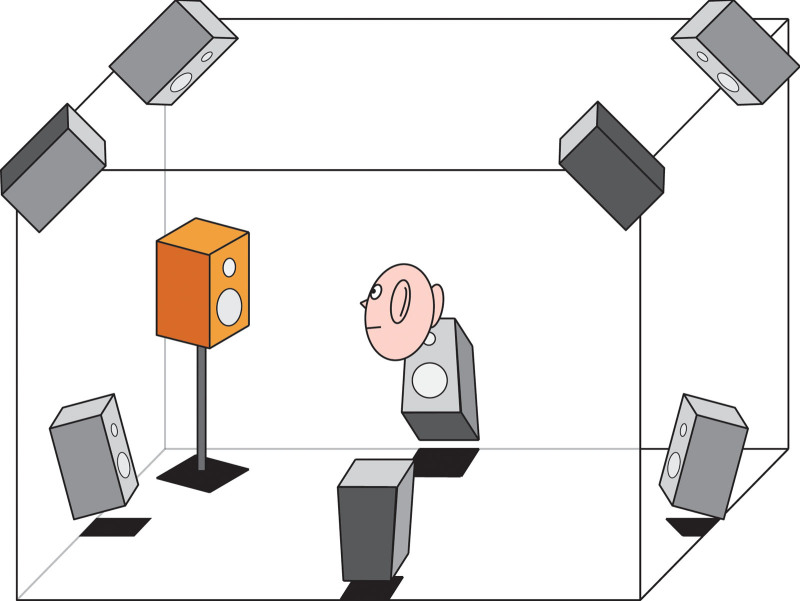
Loudspeaker configuration used to generate a homogenous field of eight-talker babble. Speech was presented in front of the listener (orange loudspeaker). Figure reproduced from Stronks et al. (2023) under the Creative Commons Attribution-NonCommercial-4.0 International License (CC BY-NC).

Testing was performed in a sound-attenuated booth measuring 3.4 × 3.2 × 2.4 m (l × *w* × *h*). The participant was seated in the middle, and noise was delivered through eight loudspeakers (Control 1; JBL Corp., Los Angeles, CA) distributed around the listener to yield a homogeneous noise field. Four of the loudspeakers were positioned in the top corners of the booth, and the others were placed close to the ground (Fig. 1). Each loudspeaker was calibrated individually to yield an identical sound level in the middle of the room to generate an 8-talker babble with a long-term average of 60 dBA. This setup has been described in more detail previously (Stronks et al. 2020, 2022). Speech stimuli were presented with a loudspeaker (MSP5A monitor speaker; Yamaha Corp., Japan) placed 1 m in front of the listener. Calibration was performed using a sound level meter (Rion NA-28; Rion Co. Ltd, Tokyo, Japan) in the middle of the room.

### Speech Recognition With the Matrix Test

CI Participants were supplied with a research speech processor (Q90; Advanced-Bionics LLC, Valencia, CA) on the day of testing. It was fitted with their own threshold and maximal comfortable stimulus levels, but all front-end processing strategies (e.g., beamformers) were turned off. Participants with additional hearing devices (hearing aids, contralateral routing of signals) were asked to remove them during testing.

Speech recognition was assessed using the speech corpus of the Dutch/Flemish Matrix test (Luts et al. 2014). The Matrix test is useful for measuring speech recognition ability and listening effort using a subjective rating scale (Stronks et al. 2020, 2021a, b). The speech corpus of this test comprises sentences of five words with a fixed grammatical syntax. Each of the 13 different available lists consisted of 20 unique sentences, and speech performance is expressed as the percentage correct of verbally repeated words.

Pupillometry was performed at four fixed SNRs that were determined for each participant individually using their individual speech recognition threshold (SRT). The SRT was defined as the SNR at which word recognition equaled 50%. The SNRs tested were set relative to SRT, namely SRT+0 dB, SRT+5 dB, and SRT+10 dB. The fourth condition was listening in quiet, corresponding to an infinite SNR. The four listening conditions are denoted as 0 dB re SRT, +5 dB re SRT, +10 dB re SRT, and quiet. The 0-dB re SRT condition was the most challenging SNR, and quiet the least. By taking the SRT as the starting point for the SNRs, as opposed to using a fixed set of SNRs for all participants, we ensured that the task was demanding, yet manageable for each participant in both groups.

The SRT was determined by adaptively varying speech level in a homogeneous field of 8-talker babble noise presented at 60 dBA, as described in detail elsewhere (Stronks et al. 2020). The adaptive rule used was described by Eq. 9 in Brand & Kollmeier (2002). The SRT was defined as the average SNR across the last 8 trials, including the SNR of the “virtual” 21st trial based on the outcome of the last trial (Francart et al. 2017).

By taking the SRT as a basis, we varied SNR by lowering the background noise from 60 to 55 dBA and 50 dBA (+5 dB and +10 dB re SRT, respectively), and by measuring in quiet (SNR = ∞). We also varied the SNR by increasing the speech level by 5 dB (+5 dB re SRT) and 10 dB (+10 dB re SRT). This procedure yielded a total of six listening conditions and four different SNRs. The +5 and +10 dB re SRT conditions were thus tested twice, once by decreasing noise level and once by increasing speech level. This enabled us to compare speech-varied and noise-varied responses. Primary outcomes were pupil dilation and subjective ratings, and word correct scores were taken as a secondary measure.

Typically, two adaptive training tests were provided at the start of the session to reduce learning effects (Stronks et al. 2020). During these 2 tests, the participants were encouraged to use a physical list containing the matrix with the 50 possible words. One training test was applied in quiet and the other in eight-talker babble noise. After these two practice trials, the participant handed back the list with the matrix words and was then instructed to fixate on the loudspeaker in front of them that produced the speech. The SRT was determined next, using the SRT obtained during practice as the starting SNR. When the measurements were obtained at the end of a test session, generally just one practice test in noise was provided to allow accommodation of the participant to wearing the glasses and the pupillometry protocol. CI users participated in experiments also involving the Matrix test that were not part of this study. Despite such precautions, the Matrix test has been associated with significant learning effects across trials and between sessions (De Jong et al. 2019).

Speech tests were carried out using custom-built software in a MATLAB R2017b programming environment (MathWorks, Inc., Natick, MA, USA). Participants were instructed to verbally repeat every word they heard after the presentation of each sentence. The experimenter recorded the answers manually. Guessing was allowed. Test conditions were randomized within and across participants. Speech recognition scores were transformed into rationalized arcsine units (Rau) to allow for quantitative statistical analysis (Studebaker 1985).

### Pupillometry

Listening effort was measured at different SNRs using pupillometry and subjective ratings. Pupil diameter was recorded during speech testing with eye-tracking glasses (ETG 2.6; SensoriMotor Instruments, Teltow, Germany). The ETG system used an infrared camera to measure pupil diameter at a spatial resolution of 1280 × 960 pixels, and a sample rate of 120 Hz. It converted the pupil size expressed in pixels to one in millimeters using a model of the eye globe.

To ensure that the room lighting was appropriate to allow both pupil dilation and constriction, the dynamic range was determined at 5 different light intensities between 40 and 540 lx (Sonel LXP-2; Świdnica, Poland) using dimmable fluorescent lights. Recordings were performed at a luminance where the pupil response was approximately in the middle of its dynamic range, and preferably in the reported mid-dynamic range of 4.5 to 5.0 mm (Watson & Yellott 2012). For 1 participant, the lights were also switched off to obtain a proper upper range of the pupil diameter.

The experimental paradigm for pupillometry and subsequent signal processing was based on the procedures reported by Zekveld et al. (2010, 2011). Each of the 20 sentences in the Matrix test was preceded with a baseline recording. Subsequently, the sentence was presented, lasting approximately 2 sec. After a period of 3 sec of only background noise, a probe tone was presented to inform the participant they were allowed to respond.

In the first 10 CI users, 4 sec of noise-only presentation preceded the sentence to allow the pupil to constrict to baseline level. This was increased to 8 sec for subsequent participants, based on circumstantial evidence of more pronounced pupil responses at longer baselines. The TH group was tested with a baseline of 8 sec.

The first 10 CI users (CS09 to CS18) were tested in a single test session where only 3 of the 6 listening conditions were tested, namely 0, +5, and +10 dB re SRT where speech was varied. In subsequent experiments with 16 CI users (SR03 to SR18), all 6 listening conditions were tested. These participants were tested in two sessions, on two separate days, yielding a test and retest of all six conditions. A retest was deemed useful, because of the variability in the data of the CI group. Three CI users in the first group of 10 participated again in the second group of 16, yielding a total of 23 CI users. All test and retest data were averaged. From the 7 CI users in the first group that did not participate in the second group, the quiet condition was lacking.

Although most of the TH participants (n = 15) were tested in a single session, 3 were tested in two sessions on different days. All six listening conditions were tested once for the TH group. The magnitude of the pupil response was generally larger and more reliable for this group of mostly young participants, and a retest was deemed unnecessary. The duration of the test sessions were approximately 2 to 3 hr each, including at least 1 break of 10 min.

Pupil dilation was recorded and analyzed for both eyes. Eye blinks were detected as intervals where pupil diameters were less than 2 mm and eliminated by linear interpolation between 10 samples before the start of the blink to 10 samples after it. Pupil sizes exceeding 8 mm were treated as artifacts, because they are outside the normal dynamic range (Watson & Yellott 2012) and eliminated via interpolation as well. After deblinking, the signal was low-pass filtered using a sixth-order Butterworth filter with a cutoff frequency of 5 Hz. The average diameter during the last second of the baseline recording was defined as the baseline diameter to determine the pupil response.

Sweeps were discarded after deblinking when any of the following criteria were met: (1) when 50%, or more, of the baseline samples were interpolated, that is, ≥0.5 sec from the available 1 sec; (2) when 33% or more of the post-baseline recording was interpolated (≥4 sec from the available 12 sec); (3) when there was an uninterrupted stretch of interpolation present that exceeded 0.5 sec. The remaining accepted traces were baseline-corrected to reduce signal drift. A last artifact rejection step was introduced to reject traces with gross residual artifacts, notably those from partially removed eye blinks, by eliminating traces with an overall amplitude (maximum minus minimum of the entire sweep) larger than the mean overall amplitude + 2·SD of the ensemble average. Subsequently, the remaining traces were ensemble averaged to reduce background noise. If the total number of included waveforms from both eyes was less than 5, the condition was discarded. In the final data set, there were two such instances of missing data. Both instances were observed in the data from CI users where a test and retest were available, and hence no missing data were generated. The smallest number of trials included in a listening condition for a single eye was 7, and for both it was 10; the latter was reached in a recording of a subject where 1 eye was discarded, and 10 trials were included for that eye. Overall, such small numbers were rare. On average, 15 and 17 sweeps were included per eye for the CI and TH group, respectively. In these averages, the discarded eyes were counted as 0, that is, the number of sweeps available in only the included eyes was larger, namely 18 sweeps per eye on average, adding up to 36 traces per pupil response. Ensemble averaged waveforms were visually inspected, and all included traces were acceptable in terms of remaining artifacts.

**Fig. 2.**
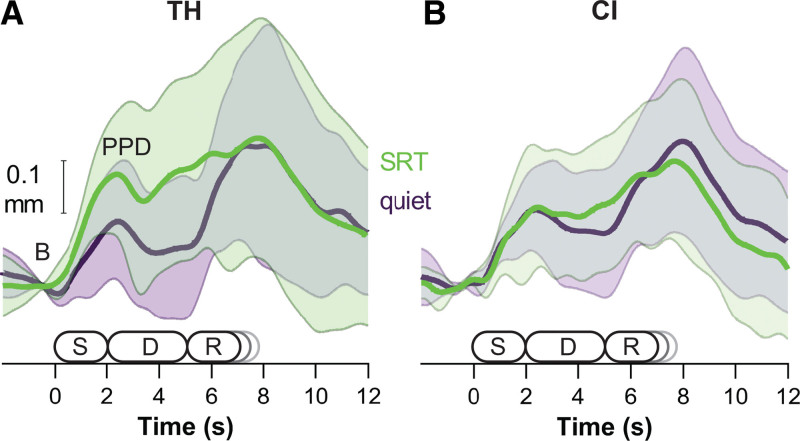
Sample pupillometric waveforms. A, TH listeners and (B) CI users when listening in background noise at a signal to noise ratio equaling 0 dB re SRT (green curve) and at the same speech level in quiet (purple curve). The abscissa shows the time in seconds. S: stimulus sentence (approximately 2 sec). D: response delay (3 sec); R: response time (approximately equal to stimulus but participant-dependent). Thick lines represent the group average, and the error margins are the SD of the group. Response amplitude was defined as the difference between the base (B) and the first peak after sentence presentation (PPD). Eight-talker babble was presented continuously during speech in noise testing. CI indicates cochlear implant; PPD, peak pupil dilation; SRT, speech recognition threshold; TH, typical hearing.

Peak pupil dilation (PPD) was calculated as the difference between the baseline pupil diameter and the maximum pupil diameter during the time period between 1 and 4 sec after the baseline period. PPD reflects the maximal listening effort during sentence presentation (Winn et al. 2018b). The second peak, occurring during the verbal response, showed a high within and between-participant variability (Fig. 2) and was not analyzed. PPDs from both eyes were averaged using equal weights.

Because the CI users were a representative sample of the CI population in our medical center, their mean age was substantially higher than the predominantly young TH controls. We corrected for this age difference by taking the smaller range of pupil sizes observed in older eyes into account (Zekveld et al. 2011) by expressing pupil dilation as a percentage of the dynamic range. The dynamic range was determined by the difference of the pupil diameter at the dimmest and highest light intensity, according to Piquado et al. (2010):

$$\begin{array}{l} R\%=\frac{R}{dmax-dmin}\cdot 100, \end{array}$$ (1)

where *R*% is the age-corrected pupil diameter (% dynamic range), *R* is the pupil response (mm), and *d*max and *d*min are the maximum and minimum pupil diameter under low and high illuminance, respectively. This correction applies to age-related differences in the physiology of the pupil but does not consider age effects on higher brain processes, such as speech processing, cognition, or working memory.

### Subjective Listening Effort and Performance Rating

After completing a Matrix test (i.e., 20 sentences at a specific SNR) where pupil diameters were recorded, participants subjectively rated their listening effort and performance on a 9-point Likert scale (Zekveld et al. 2011; Stronks et al. 2021a), as follows (English translations): no effort (1), low effort (3), moderate effort (5), high effort (7), and very high effort (9). Participants were encouraged to rate only listening effort and to ignore overall task difficulty, fatigue, or amount of concentration needed. Labels and ratings for rating performance in speech recognition were as follows: none of the words were correct (1), approximately 25% correct (3), approximately 50% correct (5), approximately 75% correct (7), and all words were correct (9). Numbers in between the labeled ones could be used as well.

### Statistical Analysis

For 7 participants for the CI group, the quiet condition was not measured, and PPDs were occasionally missing due to low data quality. Because of the resulting presence of missing values, standard repeated measures analysis of variance (ANOVA) could not be used. Therefore, statistical testing was performed using the mixed procedure of IBM SPSS Statistics for Windows (version 29.0, released 2022; IBM Corp. Armonk, NY). Linear mixed models (LMM) were constructed with the fixed factor(s) of interest (e.g., SNR, group [TH or CI], or speech/noise-varied SNR), and participant label was always included as random factor. A scaled identity covariance structure for the repeated fixed effects and random factor was used. This choice was based on a comparison of the Akaike and Bayesian information criteria (AIC and BIC, respectively) across various often-used structures for repeated measures (scaled identity, unstructured, diagonal, compound symmetry, and heterogeneous compound symmetry) using the LMM structure for the main research question, namely whether PPD was dependent on SNR, group (IC or TH), and their interaction (Eq. 3). LMMs were fitted with the restricted maximum likelihood procedure (the default) and any other LMM settings not made explicit here were left at their defaults as well, per SPSS version 29.0. Significance levels of the main (interaction) effects of the LMM were determined using *F* tests, and post hoc multiple comparisons testing was performed using Šídák’s or Bonferroni correction. The statistical outcomes of the post hoc tests between pairs of conditions are reported via the estimated marginal (EM) mean difference between the pair, the 95% confidence interval (CI_95%_) of that difference, and the associated *p* value in the form of EM mean difference (CI_95%_, *p*).

## RESULTS

### Pupil Response Waveforms

To compare pupil responses between CI users and TH listeners, we recorded their dilations as a function of SNR. Figure 2 shows the pupil response traces averaged across TH listeners (Fig. 2A) and CI users (Fig. 2B) in two different example conditions, namely in quiet (purple trace) and for the 0 dB re SRT condition with 60-dBA babble noise (green trace).

### Effect of Varying Noise or Speech Level to Adjust the SNR

**Fig. 3.**
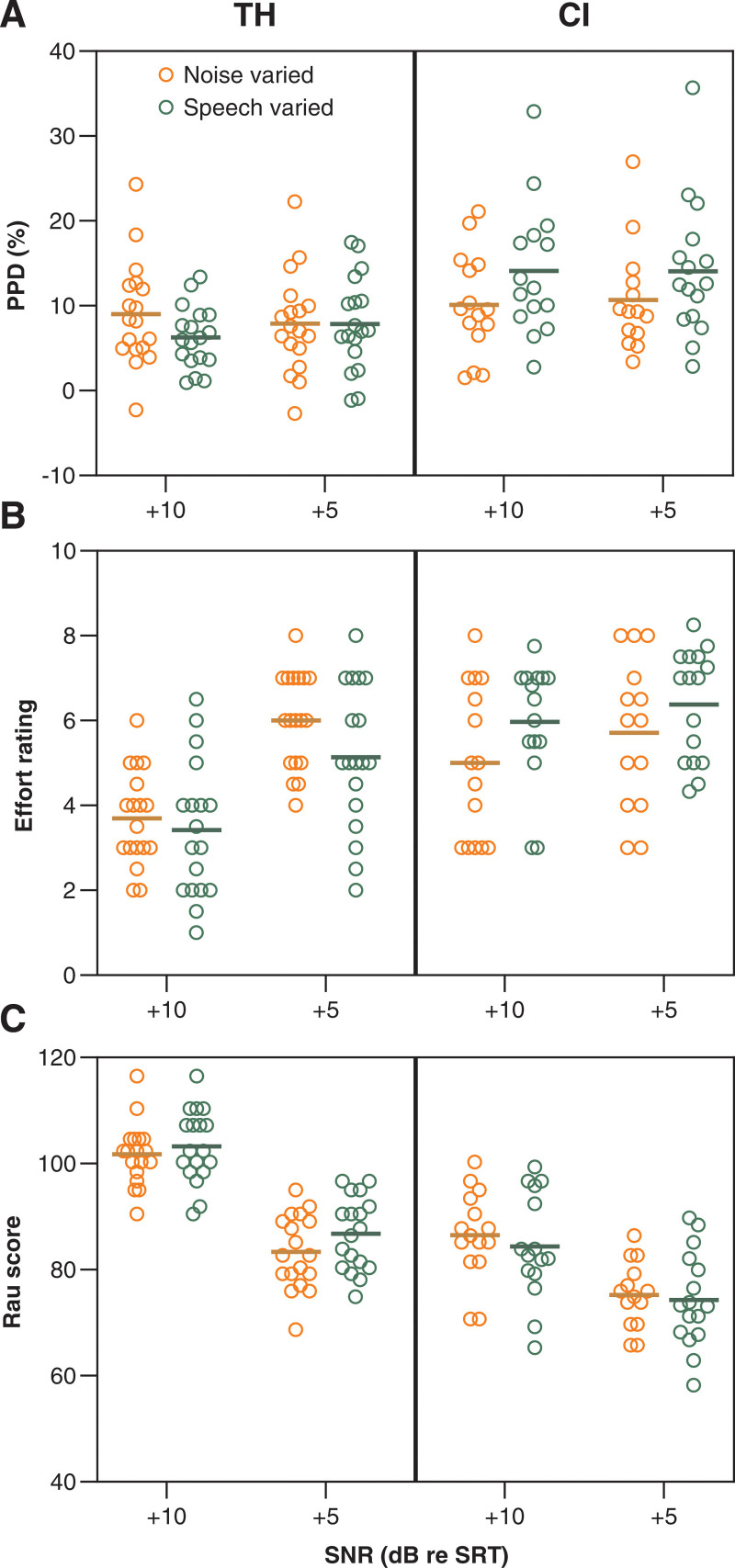
Dependence of PPD and other outcome measures on variable noise (N) or speech levels (S) to set the SNR. TH listeners are plotted left and CI users on the right in each panel. A, PPDs recorded at noise-varied SNRs (orange symbols) and speech-varied SNRs (green symbols) by reducing noise level by 10 dB (+10) and 5 dB (+5) and increasing the speech level by 10 dB (+10) and 5 dB (+5) relative to the SRT, respectively. Speech-varied PPDs were significantly larger than those obtained at noise-varied SNRs for the CI group (*p* < 0.05). B, Effort ratings at speech-varied SNRs were 0.6 points lower in TH and 0.8 points higher in CI than at noise-varied SNRs (*p* < 0.05). C, Rau scores were slightly higher at speech-varied SNRs in the TH group (*p* < 0.05). CI indicates cochlear implant; PPD, peak pupil dilation; SNR, signal to noise ratios; SRT, speech-recognition threshold; TH, typical hearing.

SNRs were established relative to the participant’s SRT by either increasing speech level by 5 and 10 dB with a fixed noise level (60 dB SPL), or by lowering the level of the multitalker babble noise by 5 and 10 dB and keeping the speech level fixed. We tested whether the PPD and the other outcome measures differed between speech-varied and noise-varied SNRs (Fig. 3). All participants of the TH group were included in the analysis (n = 18), and from the CI group only those who were subjected to both speech and noise-varied SNRs (n = 16). The other 2 SNRs (quiet, SRT) were omitted from this analysis, because they had no noise/speech-varied counterpart. The average SRT ±SD for the TH listeners was −10 ± 2 dB and for the CI users +5 dB ± 5 dB. To investigate the effect of speech and noise-varied SNRs and their interaction with group and SNR, the LMM was created for each of the outcome measures:

$$\begin{array}{l} y∼N/S+SNR+TH/CI+\left(N/S*SNR\right)\\ +\left(N/S*TH/CI\right)+\left(TH/CI*SNR\right)\\ +\left(N/S*SNR*TH/CI\right)+1|participant, \end{array}$$ (2)

where *y* is a dependent variable (PPD, ratings or Rau score); SNR and N/S are repeated fixed factors representing the signal to noise ratio (+5 or +10 dB re SRT), and whether the SNR was generated by varying noise or speech, respectively; TH/CI is a fixed factor indicating the TH or CI group; participant is the random effect factor; * indicates an interaction factor.

No significant main effect of noise and speech-varied SNRs (N/S) on PPD was found [Fig. 3A, *F*(1,93) = 1.8, *p* = 0.183]. However, a significant difference between TH and CI participants [TH/CI: *F*(1,32) = 6.6, *p* = 0.015] was found as well as a significant N/S*TH/CI interaction [*F*(1,93) = 10.9, *p* = 0.001]. No main effect of SNR nor of its interaction factors were found [SNR: *F*(1,92) = 0.1, *p* = 0.720; N/S*SNR: *F*(1,93) = 0.8, *p* = 0.372; SNR*TH/CI: *F*(1,92) = 0.0, *p* = 0.971; N/S*SNR*TH/CI: *F*(1,92) = 1.0, *p* = 0.312]. Based on the significant N/S*TH/CI term, a multiple comparisons, Šídák’s corrected post hoc test was conducted comparing PPDs between noise and speech-varied SNRs for the TH and CI groups, while disregarding any SNR effects. For the CI group, EM mean PPDs were 3.3% (1.2 to 5.4%, *p* = 0.002) larger for speech-varied, noise-fixed SNRs, whereas no significant effect was found for the TH group (1.4% larger PPDs for noise-varied, speech-fixed SNRs, −0.5 to 3.3%, *p* = 0.146).

A similar result was obtained for the effort ratings (Fig. 3B); there was no significant main N/S effect [*F*(1, 93) = 0.4, *p* = 0.535], but a significant main effect of TH/CI [*F*(1,32) = 9.1, *p* = 0.005] and a significant interaction effect between N/S and TH/CI [*F*(1,93) = 14.7, *p* < 0.001] were observed. In contrast with the PPD, SNR [*F*(1,92) = 53.5, *p* < 0.001] and SNR*TH/CI were significant as well [*F*(1,92) = 16.9, *p* < 0.001]. N/S*SNR [*F*(1,93) = 2.2, *p* = 0.146] and N/S*SNR*TH/CI [*F*(1,93) = 0.0, *p* = 0.860] were not significant. Based on the significant interaction between N/S and TH/CI, Šídák’s corrected multiple comparisons were performed identical to the post hoc tests carried out for PPD. For the TH group, effort ratings were 0.6 (0.1 to 1.0, *p* = 0.019) points lower (i.e., less effortful listening) across SNRs for speech-varied, noise-fixed SNRs. By contrast, ratings were 0.8 (0.3 to 1.3, *p* = 0.003) points higher (more effortful) for speech-varied, noise-fixed SNRs in the CI group.

The statistical outcomes of the Rau scores (Fig. 3C) mirrored those of the effort ratings; there was no significant main N/S effect [*F*(1,92) = 0.0, *p* = 0.889], and TH/CI [*F*(1,31) = 36.4, *p* < 0.001], N/S*TH/CI [*F*(1,92) = 7.969, *p* = 0.006] and SNR [*F*(1,91) = 289.1, *p* < 0.001] were all significant, but no significant effects of N/S*SNR [*F*(1,92) = 1.3, *p* = 0.257], or N/S*SNR*TH/CI [*F*(1, 92) = 0.0, *p* = 0.974] were observed. Post hoc testing showed slightly higher Rau scores for speech-varied, noise-fixed SNRs in the TH group (2.5 points, 0.3 to 6.6, *p* = 0.031), whereas in the CI group speech-varied, noise-fixed SNRs were associated with lower scores (2.2 points, −0.2 to 4.7), but not significantly (*p* = 0.074).

### Effect of Noise Level on the Pupil Baseline Diameter

PPDs were determined relative to the baseline pupil diameter. This baseline was determined in the presence of background noise, because the multitalker babble was presented continuously during testing. We investigated whether background noise affected the baseline pupil diameter with an LMM, again using the data from the subpopulation of 16 CI users where noise-varied SNRs were used, and the 18 TH listeners using an LMM. The LMM used was:

$$\begin{array}{l} baseline∼L+TH/CI+\left(L*TH/CI\right)+1|participant, \end{array}$$ (3)

where baseline is the pupil diameter when only background noise was present; *L* is a repeated fixed factor representing noise level (quiet, 50, 55, and 60 dB SPL); TH/CI is a fixed factor indicating the TH or CI group; participant is the random effect factor; * indicates an interaction factor. Noise level did not affect the baseline pupil diameter [*F*(3,93) = 0.6, *p* = 0.622] and the interaction factor was not significant either [*F*(3,93) = 0.6, *p* > 0.627]. A significant main effect of TH/CI was found [*F*(1,32) = 6.5, *p* = 0.016], however, showing that the baseline pupil size for the TH listeners was 0.8 mm (0.2 to 1.4 mm, Šidák’s corrected *p* = 0.016) larger than for the CI users. The age difference between the groups was the most likely cause of this difference (Zekveld et al. 2011).

### Effect of SNR on PPD, Effort Rating, Correct Rating, and Word Score

The remaining analyses were carried out after averaging the data pairs obtained with noise and speech-varied SNRs. The effects of varying noise or speech were small and this manipulation was not performed for all SNR conditions. As a result, collapsing the data simplified the analyses with little effect on the outcomes.

**Fig. 4.**
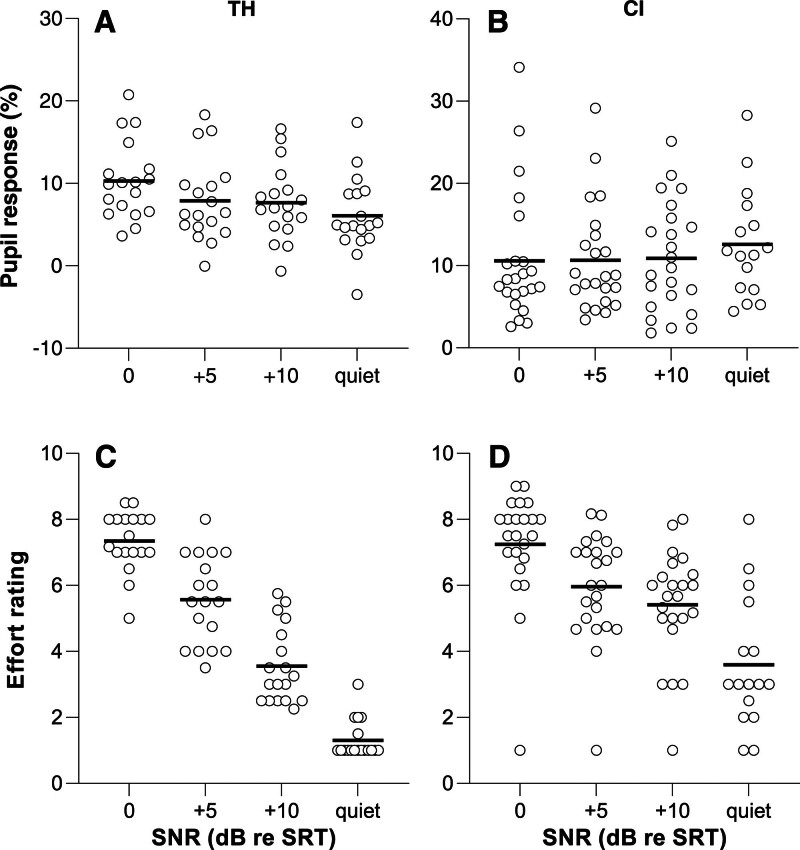
Scatter plots of the pupil responses (A, B) and effort ratings (C, D) as a function of signal to noise ratio in a group of TH listeners and CI users. The listening conditions run from challenging at SRT (0 dB re SRT), to less demanding (SNRs of +5 and +10 dB re SRT), to the least demanding (quiet). Horizontal line in each condition: average across participants. PPDs differed between quiet and 0 dB re SRT (*p* < 0.05) in TH listeners. PPD was not significantly dependent on SNR in CI users (B); ratings differed significantly from 0 dB re SRT in all other SNR conditions in both the TH and CI group (*p* < 0.001). CI indicates cochlear implant; PPD, peak pupil dilation; SNR, signal to noise ratios; SRT, speech-recognition threshold; TH, typical hearing.

Figure 4 shows PPD amplitudes, expressed as percentage of dynamic range, for TH listeners (Fig. 4A) and CI users (Fig. 4B) as a function of SNR. For TH users, PPDs were maximal at 0 dB re SRT and decreased at higher SNRs. For the CI group, the effects of SNR were less pronounced and, if anything, a pattern opposite to that observed for the TH listeners emerged. The pupil responses not corrected for dynamic range showed similar SNR-dependency, but the magnitude of the uncorrected pupil responses was on average larger for the TH group than for the CI group (results not shown).

To test the SNR effects on PPD and the secondary outcome measures for significance, the following LMM structure was used:

$$\begin{array}{l} y∼SNR+TH/CI+\left(SNR*TH/CI\right)+1|participant, \end{array}$$ (4)

where *y* is a dependent variable (PPD, ratings or Rau score); SNR is a repeated fixed factor representing the signal to- noise ratio (0 dB, +5 dB and +10 dB re SRT, or quiet); TH/CI is a fixed factor indicating the TH or CI group; participant is the random effect factor; * indicates an interaction factor.

This LMM structure was used to address the main research question, namely whether the PPD depended on task difficulty (SNR), if it differed between TH and CI, and whether the SNR effect differed between these two groups. We found no significant main effect of SNR on PPD [*F*(3,110) = 2.1, *p* = 0.107], nor of TH/CI on PPD [*F*(1,39) = 2.8, *p* = 0.100], but a significant interaction between the 2 was found [*F*(3,110) = 3.0, *p* = 0.032]. Based on this interaction , post hoc testing was performed by comparing SNRs within each group and using 0 dB re SRT as a reference (i.e., the most demanding condition tested where speech recognition was 50%). Šidák’s correction for multiple comparisons was applied. For the TH group, PPDs were 4.2% (1.5 to 6.9%, *p* < 0.001) larger at 0 dB re SRT than in the quiet condition. The EM mean difference of the PPD between the 0 dB re SRT and +10 dB re SRT (2.7%, (−0.04 to 5.3%), *p* = 0.054) and +5 dB re SRT (2.4% [−0.3 to 5.1%], *p* = 0.090) conditions were not significant. For the CI group, no significant differences were found in any of the three post hoc comparisons made (corrected *p* values were >0.9 in all cases).

The SNR dependence of the subjective effort ratings is shown in Figure 4C (TH) and Figure 4D (CI). In contrast to PPD, both factors and their interaction significantly affected effort ratings (SNR: *F*(3,109) = 195.1, *p* < 0.001; TH/CI: *F*(1,38) = 7.1, *p* = 0.011; SNR*TH/CI: *F*(3,109) = 13.7, *p* < 0.001). Šidák’s multiple comparison testing revealed that ratings at +5 dB, +10 dB re SRT, and quiet all significantly differed from 0 dB re SRT for both the TH and CI groups. The EM mean rating for TH listeners decreased by 1.8 (1.0 to 2.5, *p* < 0.001), 3.8 (3.1 to 4.5, *p* < 0.001) and 6.0 points (5.3 to 6.8, *p* < 0.001), respectively, and for CI users by 1.3 (0.6 to 1.9, *p* < 0.001), 1.8 (1.2 to 2.5, *p* < 0.001), and 3.8 points (3.1 to 4.6, *p* < 0.001) on a 1 to 9 Likert scale.

**Fig. 5.**
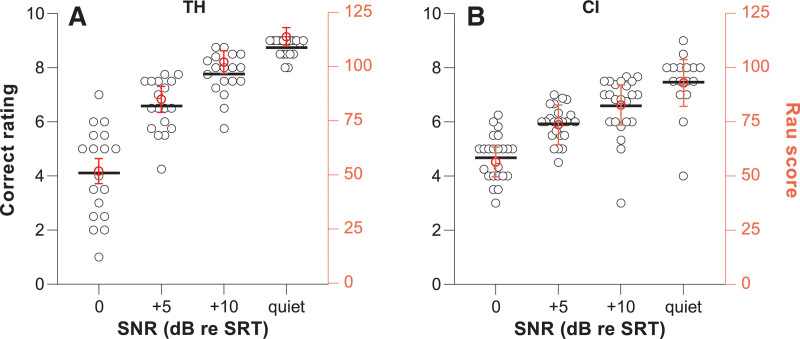
Scatter plots of the subjective correct ratings (black circles plotted on left y axis) and mean correct scores converted to a Rau scale (±SD, solid red circles plotted on the right y axis) as a function of signal to noise ratio. A, TH listeners (B) CI users. SNRs run from demanding (0 dB re SRT), to less demanding (+5 and +10 dB re SRT), to listening in quiet. Ratings and Rau scores differed significantly from 0 dB re SRT in all other SNR conditions in both the TH and CI group (*p* < 0.001). CI indicates cochlear implant; SNR, signal to noise ratios; SRT, speech-recognition threshold; TH, typical hearing.

The SNR dependence of the correct ratings (black open circles) and Rau score (mean ± SD, solid red circles) are shown in Figure 5A (TH) and Figure 5B (CI). Subjective correct ratings depended significantly on SNR [*F*(3,111) = 155.4, *p* < 0.001], TH/CI [*F*(1,39) = 7.7, *p* = 0.008], and SNR*TH/CI [*F*(3,111) = 12.1, *p* < 0.001]. Šidák’s post hoc testing against 0 dB re SRT showed that the TH and CI group both rated their perceived performance significantly higher (better) at more favorable SNRs. At +5 dB re SRT, TH listeners rated their performance 2.5 points (1.8 to 3.1, *p* < 0.001) higher, at +10 dB 3.7 points (3.0 to 4.3, *p* < 0.001), and in quiet 4.6 points (4.0 to 5.3, *p* < 0.001). CI users rated their scores on average 1.3 (0.7 to 1.8, *p* < 0.001), 1.9 (1.4 to 2.5, *p* < 0.001), and 2.7 points (2.1 to 3.3, *p* < 0.001) higher than at 0 dB re SRT, respectively.

SNR [*F*(3,110) = 641.0, *p* < 0.001], TH/CI [*F*(1,38) = 38, *p* < 0.001], and SNR*TH/CI [*F*(3,110) = 53, *p* < 0.001] also affected Rau scores. Post hoc testing against 0 dB re SRT showed significant differences with all other SNRs for both groups. As expected, word recognition scores were approximately 50% on average at 0 dB re SRT, and higher at more favorable SNRs. For TH listeners, EM mean Rau scores increased by 33 (29 to 37, *p* < 0.001), 50 (46 to 54, *p* < 0.001), and 62 (58 to 66, *p* < 0.001), and for CI users by 17 (13 to 21, *p* < 0.001), 26 (23 to 30, *p* < 0.001), and 36 (31 to 40, *p* < 0.001) at +5 dB and +10 dB re SRT and in quiet, respectively.

### Correlation Between PPD and Subjective Rating of Listening Effort

**Fig. 6.**
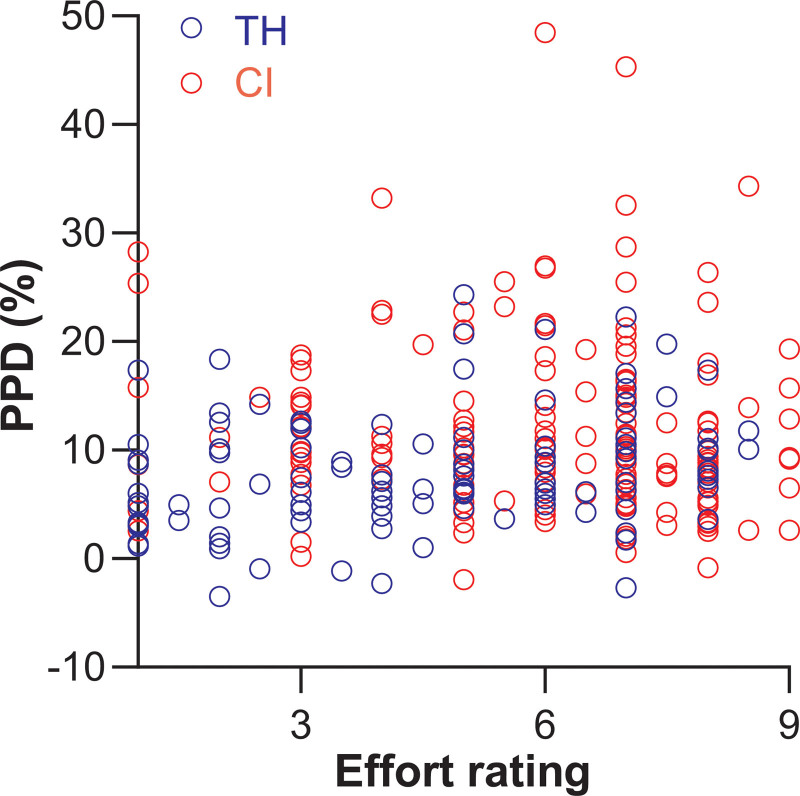
Correlation between PPD and effort rating in CI users (red symbols) and TH listeners (blue symbols). Linear mixed modeling showed no significant dependence of PPD on subjectively perceived effort. CI indicates cochlear implant; PPD, peak pupil dilation; TH, typical hearing.

In TH listeners, both PPD and subjective ratings of listening effort depended significantly on task difficulty (SNR), whereas for CI users only ratings were significantly influenced by SNR. The correlation between subjective and objective listening effort was investigated by plotting PPDs against their corresponding effort rating (Fig. 6), and tested for significance by adding subjective effort ratings as a covariate to Eq. 4 to the following full factorial model:

$$\begin{array}{l} PPD∼EffortRating+SNR\\ +TH/CI+\left(EffortRating*SNR\right)\\ +\left(EffortRating*TH/CI\right)+\left(SNR*TH/CI\right)\\ +\left(EffortRating*SNR*TH/CI\right)+1|participant, \end{array}$$ (5)

where PPD is the peak pupil dilation; EffortRating is a fixed covariate representing the subjectively rated listening effort; SNR is a repeated fixed factor representing the signal to noise ratio; TH/CI is a fixed factor indicating the group; participant is the random effect factor; * indicates an interaction factor. PPD did not depend significantly on subjective effort [*F*(1,132) = 1.888, *p* = 0.172] and there were no significant interaction effects between ratings of effort and any of the other variables (EffortRating*SNR: *F*(3,107) = 0.7, *p* = 0.554; EffortRating*TH/CI: *F*(1,132) = 0.0, *p* = 0.980; EffortRating*SNR*TH/CI: *F*(3,107) = 2.4, *p* = 0.073). SNR, TH/CI and their interaction were investigated with Eq. 4.

### Role of Subjective Versus Objective Performance Measures in Outcomes

**Fig. 7.**
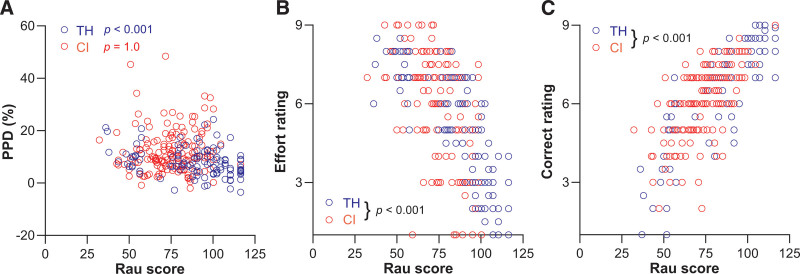
Correlations between word correct scores, expressed on a RAU scale and objective and subjective measures of effort and performance for CI users (red symbols) and TH listeners (blue symbols). A, PPD, (B) subjective effort, and (C) subjective performance as a function of Rau score. Outcomes of linear mixed modeling are shown for each group individually when the interaction of group and Rau score was significant (A) or an overall *p* value for both is provided (accolade) when the interaction did not reach significance (A, B). CI indicates cochlear implant; PPD, peak pupil dilation; RAU, rationalized arcsine units; TH, typical hearing.

Rau scores were typically better for TH listeners than for CI users at SNRs higher than 0 dB re SRT; at 0 dB re SRT they were approximately 50%, as intended. To investigate whether Rau scores affected the outcome measures for both groups differently, their correlations (Fig. 7) were tested in a similar approach as Eq. 5 by adding Rau scores to Eq. 4. However, and in contrast with Eq. 5, SNR was not included as a fixed effect, because we were specifically interested in the dependence of listening effort and perceived performance on the actual performance, regardless of the underlying cause of the difference in performance:

$$\begin{array}{l} y∼Rau+TH/CI+\left(Rau*TH/CI\right)+1|participant, \end{array}$$ (6)

where *y* is a dependent variable (PPD or effort rating); Rau is a fixed covariate representing performance; TH/CI is a fixed factor indicating the group; participant is the random effect factor; * indicates an interaction factor. PPD (Fig. 7A) did not significantly depended on Rau [*F*(1,121) = 2.3, *p* = 0.131] or TH/CI [*F*(1,148) = 1.9, *p* = 0.170], but there was a significant interaction effect between Rau and TH/CI on PPD [*F*(1,121) = 6.8, *p* = 0.010]. Post hoc analysis with Bonferroni correction showed that Rau was a significant predictor of PPD for the TH group, with an estimated slope of −0.06% PPD/Rau point (−0.09 to −0.04, *p* < 0.001). In line with the SNR dependence, PPD did not significantly depend on Rau for CI users (+0.02%/Rau point, −0.04 to 0.08, adjusted *p* = 1.0).

The correlation between effort rating and Rau score was significant [*F*(1,125) = 319.5, *p* < 0.001], but TH/CI [*F*(1,152) = 0.0, *p* = 0.875] and Rau*TH/CI [*F*(1,25) = 0.064, *p* = 0.801] had no significant effects. The slope of the TH and CI group combined was −0.09 Likert points/Rau point (0.10 to 0.08, *p* < 0.001). Similarly, the correlation between Rau score and correct rating was significant [*F*(1,132) = 456.7, *p* < 0.001], but TH/CI [*F*(1,150) = 1.3, *p* = 0.252] and Rau*TH/CI [*F*(1,132) = 0.734, *p* = 0.393] were not. The estimated slope across both groups was 0.074 [0.07 to 0.08, *p* < 0.001] Likert points/Rau point.

## DISCUSSION

In this study, the pupil responses associated with speech recognition in noise did not significantly depend on SNR nor on performance for CI users, whereas for TH listeners PPDs depended significantly on SNR and Rau scores (Figs. 4 and 7). By contrast, subjectively perceived listening effort, perceived performance and actual performance depended significantly on SNR for both groups (Figs. 4 and 5). The statistically significant dependency of speech recognition performance on SNR shows that we succeeded in adjusting task difficulty, and by taking the SRT as a starting point we ensured that all participants were able to perform the task.

We did not find a significant relation between PPD and subjectively perceived effort (Fig. 6). This discrepancy between physiological and subjective measures of listening effort supports the thought that physiological measures and subjectively perceived effort can reflect different components of cognitive load (Francis et al. 2016). Correct ratings and effort ratings correlated significantly with correct scores for both groups (Fig. 7), corroborating previous reports (Alhanbali et al. 2017), and indirectly suggesting that subjectively rated listening effort may have reflected subjectively perceived performance, rather than listening effort.

Anecdotal subjective reports from CI users in this study included a difficulty to isolate listening effort from task complexity, as the participants were explicitly instructed to do for the rating test. The current experiment was quite demanding and complex, especially for elderly participants, because: (1) they had to listen to speech in fluctuating noise, which is notoriously difficult for CI users (Stronks et al. 2020); (2) they had to remember the sentence and wait for the probe tone to sound, taxing their working memory, and then repeat the sentence back; (3) meanwhile they had to maintain their gaze during testing and keep their eyes open (blinking was allowed). Looking up when thinking is a common, automated behavior for many people, and CI participants often prefer to close their eyes during testing to maintain focus. As a result of the prerequisites for pupillometry, the test was procedurally taxing, even apart from listening under the adverse conditions in a background of babble noise. The measures of listening effort reported here (PPD, ratings) thus may have been reflections of both cognitive load due to the test procedures and listening in noise. This may explain the discrepancy between the objective PPD and subjective ratings in the present study, because different methods tap into different modalities associated with listening effort (Alhanbali et al. 2019).

The finding that widely varying SNRs resulted in significant changes in subjectively perceived listening effort for TH listeners and CI users, but that pupil responses were affected significantly only for TH listeners can have several explanations. For the CI users, all conditions may have been effortful, such that their baseline degree of effort (in quiet) was higher even in the quiet condition and adding noise might have had little additional effect. We cannot directly test this prediction, however, because of the age difference between the groups that might have affected other factors such as cognition. Second, pupil responses for CI users have been shown to be smaller and more variable than for TH listeners, according to studies involving a lexical decision task (Wagner et al. 2019) and listening in the presence of auditory distractors (Winn & Moore 2018a). Note that the raw magnitude of the pupil response was smaller for CI users than for the predominantly young TH group (pupil diameters not shown), but that the dilations were larger only after age correction for CI users (Fig. 4). The higher pupil variability for CI users has been related to the possibility of more variable sources of effort for CI users (Wagner et al. 2019). Pupil dilation reflects cognitive processing load, which is correlated to auditory stimulus characteristics including subjective loudness, speech intelligibility, type of background noise, syntactic complexity, and spectral resolution. However, besides effortful listening, pupil dilation has also been related to motivation, fatigue, cognitive abilities, and attention (Laeng et al. 2012). Any or all these parameters may differ between CI users and (young) TH listeners.

Another potential reason for the difference between the 2 groups is the use of the Matrix test. It has been shown by others that CI users depend more on semantic context than TH listeners (O’Neill et al. 2019). In contrast with the sentences used in Wagner et al. (2019) and Winn and Moore (2018a), the Matrix test lacks semantic context. All of the sentences in this test have the same syntactic structure (Luts et al. 2014) and consist of semirandom combinations of a name (e.g., Jacob), verb (e.g., buys), numeral (e.g., 5), color (e.g., blue), and object (e.g., boats). Each word has 10 alternatives. Because of this lack of context, Matrix sentences do not allow for correction of the answer based on sentence incoherence or post diction (i.e., “filling in the blanks” through mental repair of mistakes). Mental repair and reconstruction of words have been shown to be more important to the pupil response than perceiving the target correctly or incorrectly (Winn & Teece 2022). The authors of this study showed also that sentence coherence is an important driver of listening effort and stress the importance of testing materials that do not constrain coherence by design (Winn & Teece 2021). Post diction is difficult in the Matrix test due to a lack of context. In addition, the occurrence of sentence incoherence upon verbally repeating the target by the participant is rare, because of the fixed syntactic structure of the Matrix sentences. As a result, Matrix sentences may not be the best option for use in pupillometry to measure listening effort.

These considerations should be placed in the light that participants did not have access to the word matrix during most of the testing (a necessary step to perform the pupillometry), which is a departure from the more conventional way the Matrix test is used. In our experiment, the Matrix sentences were thus a quasi-closed set, which may have caused a shift from the traditional word discrimination task to one of word recognition. This may have impacted the cognitive processing during listening.

In contrast with CI users, TH listeners showed significant effects of SNR on pupil responses in the present study, hinting at the possibility that the factors that make listening effortful differ between TH listeners and CI users. More research is needed to elucidate the effects of speech material and procedural differences on pupillometric assessment of listening effort for CI users as a function of SNR, or more generally, task difficulty.

Our aim was to investigate pupillometry as a tool to assess listening effort associated with speech recognition for CI users. For pupil dilation to be useful for comparing speech enhancement strategies or other device fittings to improve speech intelligibility in noise (e.g., bimodal fittings), listening effort should be assessed during speech recognition by CI users between listening conditions, as tested here. According to our findings, however, pupillometry with the speech corpus of the Dutch/Flemish Matrix test does not emerge as a promising method for this goal in a representative group of CI users. These results are in line with previous findings from our lab (Stronks et al. 2021a). Although this is not encouraging for the use of the Matrix test to measure listening effort for CI users, our findings corroborate earlier results that pupillometry can be useful for comparing listening effort between groups (Winn & Moore 2018a; Wagner et al. 2019). Furthermore, assessing SNR effects on pupil dilation as a measure of listening effort for TH listeners is feasible with the Matrix test. However, PPD differed significantly only in quiet and at +10 dB re SRT from 0 dB re SRT. At +5 dB re SRT, the difference in PPD was not significant. Many speech enhancement strategies, such as beamformers, may not exceed 5 dB SNR benefit (Vroegop et al. 2018; Ernst et al. 2019; Stronks et al. 2022, 2023) and studies with better statistical power (i.e., more study participants) would be needed to investigate listening effort with more subtle SNR effects.

We observed that PPDs obtained from CI users were significantly larger when speech level was varied and noise level was fixed, than when noise-varied, speech-fixed SNRS were used (Fig. 3). This effect was not seen for TH listeners and was not significantly dependent on SNR. Listening was also rated to be more effortful for CI users in the speech-varied condition. The SRT for CI users averaged to approximately +5 dB, which was 15 dB higher than the SRT for the TH group. The +5 dB and +10 dB re SRT, speech-varied, noise-fixed SNR condition for the CI group thus reached the highest speech levels encountered in this study, namely 70 and 75 dBA on average, corresponding to approximately 75 to 80 dB SPL. The corresponding noise-varied, speech-fixed conditions had average speech levels of only 65 dBA. Unpublished clinical observations for our CI population implanted with Advanced Bionics devices also show that their performance declines at speech levels of 75 dB SPL and above in quiet (results not shown), and this rollover effect has been reported by others as well (Franck et al. 2003). A plausible reason for the performance drop at high sound levels is the compression applied by the adaptive gain control in Advanced Bionics devices at these sound levels (Vaerenberg et al. 2014).

By contrast, effort ratings were significantly lower when speech was varied for TH listeners at +5 dB re SRT and, accordingly, Rau scores were slightly (but significantly) higher in this condition. This result agrees with earlier findings that the SRT is more favorable when speech-variable SNRs are deployed for TH listeners (Wilson & McArdle 2012).

This study had some specific limitations, including the age distribution of the 2 participant groups. The CI group was substantially older than the TH group and it has been shown that age can negatively affect both pupil response magnitude and speech recognition performance (Plomp & Mimpen 1979; Winn et al. 1994; Zekveld et al. 2011). We performed an age correction by normalizing the pupil response relative to dynamic range (Zekveld et al. 2011), but this correction does not capture all age-related effects, such as a decline in cognitive performance or working memory. Nonetheless, our main conclusion stands that measuring listening effort with pupillometry using the Matrix test may not be feasible for older CI users.

Learning effects between sessions and trials may have played a role as well, as expected with the Matrix test (Stronks et al. 2020, 2021a). Because the listening conditions (SNRs) were randomized, we do not expect this to have negatively influenced our findings.

Although the ratings yielded significant SNR dependencies for the CI group, the rating task was not accompanied by a standard reference with a fixed SNR (i.e., an anchor). Ratings of effort are subjective, and anecdotal reports from participants indicated they typically rated their effort using earlier tests as a reference, rather than using the last performed test only. Indeed, the order of tests and range of SNRs are known to influence ratings (Krueger et al. 2017), so that the initial rating may have determined the dynamic range in subsequent tests. Our own anecdotal observations indicated that participants who gave the initial test a high rating at a relatively favorable SNR also produced rating values at subsequent SNRs that were compressed into the small remaining dynamic range. Providing participants with an anchor at each test is expected to result in more reliable ratings (Krueger et al. 2017).

## CONCLUSION

The matrix test may not be a feasible tool for measuring within-group effects of SNR on listening effort with pupillometry for CI users to measure. In this study, a subjective rating test of listening effort was robustly and significantly dependent on the SNR for both the CI users and the TH listeners, but may have reflected speech recognition performance, rather than exerted effort. Speech-varied, noise-fixed SNRs can have subtly, yet significantly different effects on measures of listening effort than noise-varied SNRs, and these effects may differ between TH listeners and CI users.

## ACKNOWLEDGMENTS

We are grateful to the study participants for their time and dedication, and we thank Eline Apperloo for assistance during data collection.
